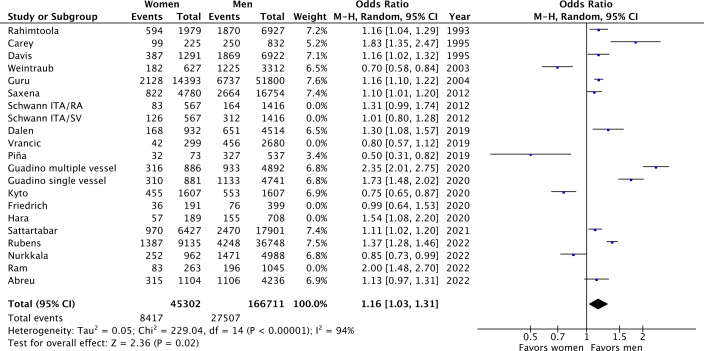# Sex-related differences in long-term mortality after coronary artery bypass graft surgery: A systematic review and meta-analysis

**DOI:** 10.1016/j.ijcrp.2026.200611

**Published:** 2026-02-26

**Authors:** David T. Zhang, Simrat Dhaliwal, Navindra Tajeshwar, Gregg A. Stevens, Mohammed Al-Sadawi, Michael Tao

**Affiliations:** a Division of Cardiology, Department of Medicine, Stony Brook Medicine, Stony Brook, NY, USA; b Division of Cardiology, Department of Medicine, Albany Medical Center, Albany, NY, USA; c University of Massachusetts Chan Medical School, Worcester, MA, USA; d Cardiovascular Division, Department of Medicine, University of Minnesota, Minneapolis, MN, USA; e Heart & Vascular Institute, Hartford Healthcare Medical Group, Hartford, CT, USA

**Keywords:** Sex, Gender, Mortality, Coronary artery bypass graft, CABG, Women, Outcomes

## Abstract

**Background:**

This meta-analysis assessed the sex-related differences in long-term mortality (≥7 years) after coronary artery bypass graft (CABG) surgery.

**Methods:**

We searched the databases Ovid MEDLINE, Embase, and Web of Science for studies reporting sex-specific differences in mortality following CABG. The search was not restricted to time or publication status. The primary endpoint of interest was long-term mortality (≥7 year or longer).

**Results:**

A total of 1289 studies resulted from literature search. A total of 19 studies with 277,224 patients (60,858 women and 216,369 men) were included. The mean age was higher in women than men (66.5 vs. 63.1 years). Our analysis demonstrated that female sex was associated with higher long-term mortality (≥7 years) following CABG (odds ratio 1.17; 95% confidence interval 1.05-1.30; p < 0.01).

**Conclusions:**

Our results suggest that women had higher long-term mortality following CABG compared with men. It is the largest study to date of sex-specific differences in long-term mortality (≥7 years).

## 1 Introduction

Cardiovascular disease (CVD) is the leading cause of death for women and men across the United States [1]. Yet, historically women have had higher rates of underdiagnosis, undertreatment, and increased rates of morbidity and mortality. Drivers of these disparities can likely be attributed to sex-based differences in pathophysiology, patient populations, disease presentation, physician, and patient education as well as differences management [2]. Over the past decade, research in the evolving field of women's CVD has helped to bridge the outcome gap [3,4]. However, questions remain on the best surgical or procedural approaches to utilize when invasive therapies are pursued [5]. Coronary artery bypass grafting (CABG) is standard of care for patients in management of complex coronary anatomy, with various factors such as sex, comorbidities, and graft type affecting outcomes [6,7]. While many valuable sex-based studies looking at CABG outcomes have been performed through the years, these works have often been limited by their retrospective nature, length of follow up, inadequate correction for confounding factors, or focus on sex only as a secondary outcome or post-hoc analysis [8,9]. Much of the literature demonstrates conflicting results [10,11].

To synthesize the impact of sex on complex coronary disease and CABG outcomes, we conducted a comprehensive meta-analysis to resolve conflicts in the literature and provide data on long-term outcomes while reflecting modern surgical techniques, practice patterns and understanding of women's cardiovascular disease.

## 2 Methods

### 2.1 Data search

The systematic review was conducted in June 2025 using a preplanned protocol in accordance with the Preferred Reporting of Items for Systematic reviews and Meta-Analyses (PRISMA) statement [12]. Databases searched included Ovid Medline, Embase, and Web of Science with respective MeSH terms: 1) (“coronary artery bypass” OR “CABG”) AND (“Sex” OR “gender”) AND (long term) AND (“mortality”) NOT (“percutaneous”) NOT (“early” OR “in-hospital”), 2) ((‘coronary artery disease'/exp OR (multivessel-coronary-artery-disease OR coronary-disease):ab,ti)) AND ((‘coronary artery bypass graft’/exp) OR ((CABG) OR ((aort∗ OR coronary) NEAR/3 (bypass OR shunt OR anastomosis OR graft)):ab,ti)) AND ((‘mortality”/exp) OR (mortality):ab,ti) AND (‘sex difference’/exp) AND ((‘female’/exp) OR (woman OR women OR female OR females):ab,ti), and 3) (“coronary artery bypass” OR “CABG”) AND (“Sex” OR “gender”) AND (“mortality”) NOT (“percutaneous”) NOT (“early” OR “in-hospital”). In addition, reference lists of selected articles were screened for relevant citations missed by electronic searches. All study designs (retrospective, prospective, registries, and randomized controlled trials) were included. The search was not restricted to time or publication status. This systematic review was conducted using a preplanned protocol developed prior to data extraction; however, the protocol was not prospectively registered in PROSPERO. The absence of registration is acknowledged and discussed as a limitation.

### 2.2 Study selection

Studies were screened independently by two investigators (DTZ and NT) at the level of title and abstract, and then full-length reports were retrieved for detailed evaluation. The articles were selected for eligibility according to prespecified inclusion and exclusion criteria. Any discrepancy was resolved by another investigator (MT). Inclusion criteria included articles reporting published data on the incidence or the number for all-cause mortality on more than or equal to 7 years follow-up, longer than the oft-reported 5-year benchmark, separately for both women and men who had CABG surgery. Exclusion criteria abstracts, review articles, data reporting mortality without stratification based on sex, or publication in languages other than English. Full text of four articles could not be obtained through Interlibrary Loan requests. As a result, those studies could not be included. Eight additional relevant articles were identified through screening of references cited in the studies located through database searching.

### 2.3 Data extraction

Data extraction was done by two investigators (DTZ and NT) and discrepancies were resolved by consensus. A table was designed to record data of eligible studies on the year of publication, study period and type, number of participants, women and men, follow-up duration, and mortality data. Missing data was not imputed.

### 2.4 Statistical analysis

Meta-analysis of summary statistics from individual studies was performed using Cochrane Review Manager (RevMan) software, Version 5. The association between sex and mortality was presented as an odds ratio (OR) with 95% confidence interval (CI) and p-value. A the random-effects model was used for meta-analysis. The extent of heterogeneity was determined by I^2^ (ranging from 0% to 100%) with 0% to 40% defined as insignificant, 40% to 60% defined as moderate, 60% to 90% defined as substantial, and 90% to 100% defined as considerable heterogeneity. Publication bias was assessed using funnel plot inspection and Egger's regression test. Statistical significance was considered with a *P*-value <0.05 and all tests were 2-sided.

### 2.5 Risk of bias/sensitivity analysis

Study quality was assessed independently by two investigators. Observational studies were evaluated using the Newcastle–Ottawa Scale (NOS), and randomized controlled trials were assessed using the Cochrane Risk of Bias tool. Disagreements were resolved by consensus.

Sensitivity analyses were performed by excluding studies classified as moderate risk of bias (NOS score ≤6) to evaluate the robustness of the pooled effect estimate. The direction and magnitude of the association between sex and long-term mortality after CABG were compared with the primary analysis.

## 3 Results

### 3.1 Literature search and study selection

**Fig. 1:**
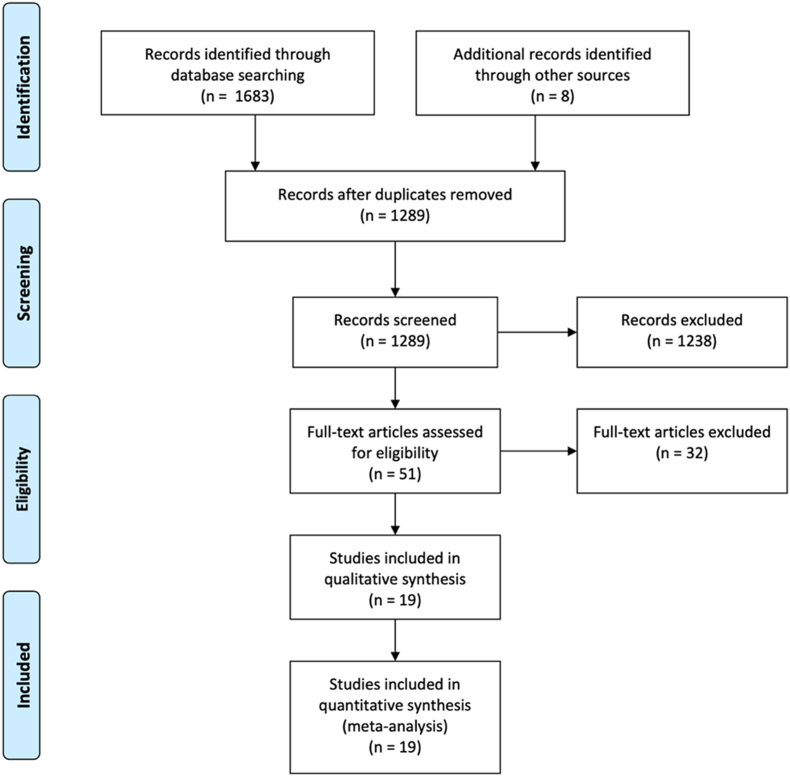
PRISMA flow diagram depicts study selection for inclusion in the meta-analysis according to the PRISMA statement for reporting systematic reviews and meta-analyses.

The primary literature search identified potentially eligible, after removing duplicates, 1289 studies. Process of study inclusion is illustrated in detail in Fig. 1. A total of 1238 citations were excluded by screening the titles and abstracts. The remaining 51 articles were examined for full‐text screening for relevance. A total of 19 articles were eligible for systematic review and meta-analysis for long-term mortality [[13], [14], [15], [16], [17], [18], [19], [20], [21], [22], [23], [24], [25], [26], [27], [28], [29], [30], [31]].

### 3.2 Study, patient, and procedural characteristics

Baseline characteristics of the included studies are presented in Table 1. The total number of patients included in the meta-analysis is 277,224 adults (60,858 women and 216,369 men), 22.0% were women, and the mean age of the women was 66.5 years and 63.1 years for men. Follow-up duration varied across studies but met or exceeded the predefined minimum of seven years.

**Table 1:** Studies comparing mortality in men versus women undergoing CABG.

Study (Year)	Study Period	Study Type	Follow-Up (years)	Number of Patients			Mean age (years)		HFrEF (%)		ACS (%)	
				Total	W	M	W	M	W	M	W	M
Rahimtoola et al. (1993)^13^	1974-91	R	17.5	8906	1979	6927	64	61	45	52	34	30
Carey et al. (1995)^14^	1972-88	R	10	1057	225	832	64.1	60.4	18	9.1	32.1	38.7
Davis et al. (1995)^15^	1972-87	RCT	15	8213	1291	6922	57.5	54.6	14	9	48	56
Weintraub et al. (2003)^16^	1973-79	R	14.2	3939	627	3312	57	54	6.7	4.8	50	54.8
Guru et al. (2006)^17^	1991-2001	R	11	66193	14393	51800	65	62	13	9	n/a	n/a
Saxena et al. (2012)^18^	2001-2009	R	7	21534	4780	16754	68.8	64.9	3.2	5	54	52.4
Schwann et al. (2012) ITA/RA^19^	1996-2007	R	7.3	1983	567	1416	65.1	62.3	12.9	8.0	36.3	30.7
Schwann et al. (2012) ITA/SV	1998-2005	R	7.3	1983	567	1416	64.8	62.3	12.0	8.3	35.3	30.0
Dalen et al. (2019)^20^	1995-2013	R	11.8	5446	932	4514	46.1	46.8	5.9	5.7	48.6	43.1
Vrancic et al. (2019)^21^	2000-17	R	9	2979	299	2680	67.8	63.8	10.7	14.4	35.8	41.8
Piña et al. (2018)^22^	2002-07	P	9.8	610	73	537	63.4	59.3	66.2	57.0	75.7	77.3
Gaudino et al. (2021) single vessel^23^	2005-14	R	7	50773	13146	37627	69.2	66.3	16.2	21.1	21.4	19.5
Gaudino et al. (2021) multiple vessels	2005-14	R	7	12629	2101	10528	64.8	60.6	11.1	13.9	18.5	17.9
Kyto et al. (2020)^24^	2004-14	R	7.1	6683	1607	5079	70.9	66.7	n/a	n/a	100	100
Friedrich et al. (2020)^25^	1998-2016	P	8	590	191	399	65.8	61.8	1.0	1.5	5.5	3.7
Hara et al. (2020)^26^	2005-07	RCT	10	897	189	708	67.9	64.2	4.4	5.6	28.0	35.4
Sattartabar et al. (2021)^27^	2007-17	R	7	24328	6427	17901	n/a	n/a	n/a	n/a	n/a	n/a
Rubens et al. (2022)^28^	2008-17	R	9	45883	9135	36748	68.1	65.5	23.7	18.3	43.8	39.1
Nurkkala et al. (2022)^29^	1998-2019	R	9.3	5950	962	4988	n/a	n/a	n/a	n/a	n/a	n/a
Ram et al. (2022)^30^	2000-16	R	8	1308	263	1045	70	63	11.8	5.5	100	100
Abreu et al. (2022)^31^	2000-15	R	12.8	5340	1104	4236	66.7	62.9	19.1	19.6	36.4	36.1

CABG = coronary artery bypass graft surgery, M = men, W = women, HFrEF = heart failure with reduced ejection fraction (left ventricular EF ≤ 40%), ACS = acute coronary syndrome; R = retrospective; P = prospective; RCT = randomized controlled trial; ITA = internal thoracic artery, RA = radial artery, SVG = saphenous vein graft.

### 3.3 Association between sex and mortality in CABG

**Fig. 2:**
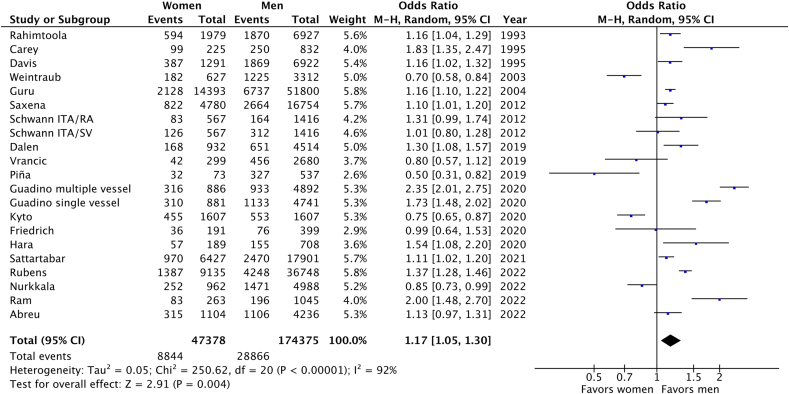
Forest plot demonstrating impact of sex on long-term outcomes following CABG.

The association between sex and long-term mortality after undergoing CABG was quantitatively studied in 19 studies [[13], [14], [15], [16], [17], [18], [19], [20], [21], [22], [23], [24], [25], [26], [27], [28], [29], [30], [31]]. The pooled results demonstrated that following CABG, women had higher long-term mortality after 7 years of follow up (OR 1.17; 95% CI 1.05-1.30; p < 0.01) compared with men (Fig. 2).

### 3.4 Publication bias

**Fig. 3:**
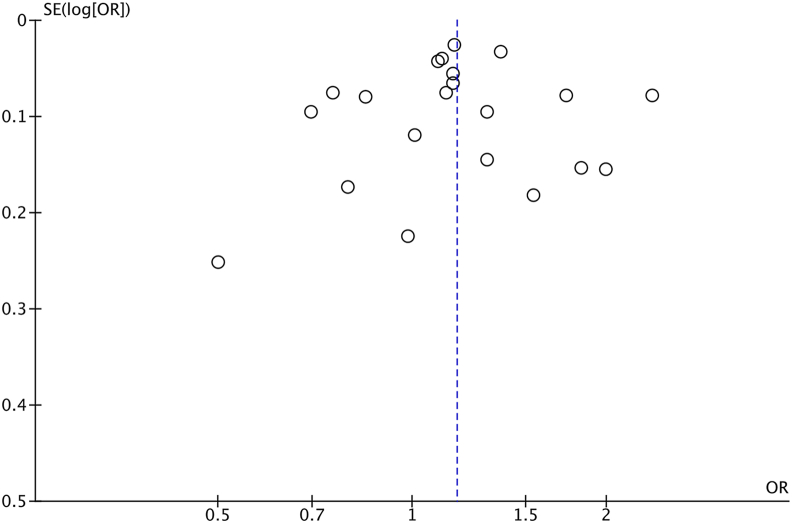
Funnel plot of included studies assessing the impact of sex on 7-year long-term outcomes following CABG.

Visual inspection of the funnel plot (Fig. 3) did not demonstrate marked asymmetry. Egger's regression test did not identify statistically significant small-study effects (intercept −0.36, p = 0.054).

### 3.5 Sensitivity analysis

Studies were classified by risk of bias (Supplemental Table 1). In sensitivity analyses excluding studies classified as moderate risk of bias (NOS score ≤6), the direction and magnitude of the association between female sex and long-term mortality after CABG remained consistent with the primary analysis (OR 1.16, 95% CI 1.03-1.31; p = 0.02) (Supplemental Fig. 1).

## 4 Discussion

This systematic review and meta-analysis represents the largest study to date examining sex-specific differences in long-term mortality following CABG, with a follow-up period of at least seven years. Our analysis revealed that women who underwent CABG had a significantly higher long-term mortality rate compared to men. The extensive scope of this study and the inclusion of both randomized and observational cohorts provide robust evidence on the subject, which could help guide clinical decision-making and highlight the need for tailored interventions for female patients undergoing CABG.

This study is the first meta-analysis to address longer-term mortality in the association between sex and patients undergoing CABG. Previous studies have shown that women have worse outcomes after percutaneous coronary angiography [4]. Moreover, similar outcomes found in shorter-term mortality after CABG, namely five years or shorter [10,32,33]. Our study extends that mortality timeframe to seven years. Taken together, these findings demonstrate an association between female sex and long-term mortality for patients undergoing CABG. However, age is a critical confounder in this association. Women undergoing CABG were older than men across most included studies, and advanced age is strongly associated with worse long-term outcomes. Because this meta-analysis relied on aggregate study-level data, adjustment for age and other covariates was not feasible, and residual confounding is possible. The substantial heterogeneity observed likely reflects differences in study era, patient selection, baseline comorbidities, operative techniques, grafting strategies, completeness of revascularization, and duration of follow-up. Inconsistent reporting of these variables precluded meaningful subgroup analyses or meta-regression.

There are some considerable biologic factors that may explain the association between sex and mortality in such population. The mean age of women in most of the registries and the studies is higher than that of men; in our meta-analysis it is 66.5 vs. 63.1 years. Women often present at older ages with a higher burden of comorbidities, smaller coronary vessel size, and higher prevalence of microvascular dysfunction, which may complicate surgical revascularization and limit graft durability [34]. Women are also less likely to receive complete revascularization or multiple arterial grafts and have lower rates of referral to and participation in cardiac rehabilitation. On the other hand, women have higher rates of readmission with congestive heart failure and myocardial infarction after CABG which may lead indirectly to higher long-term mortality [35]. Differences in postoperative medical therapy and longitudinal follow-up may further contribute to outcome disparities.

Cardiovascular rehabilitation after cardiac surgery showed improvement in short- and long-term outcomes, however, there still are sex differences in cardiovascular rehabilitation referral and enrollment [36]. Moreover, the reported association between sex and short-term mortality has steered surgeons’ decisions more toward conservative approaches in women, particularly with higher age and risk factors [37].

## 5 Limitations

Our study is the largest analysis providing sex-specific outcomes of late-term mortality in women and men after CABG. However, this study has several limitations. First, the absence of individual patient-level data precluded adjustment for key confounders such as age, comorbidities, and surgical complexity. Second, the protocol was not prospectively registered. Third, most included studies were observational, and many were not designed to evaluate sex-specific outcomes. Fourth, heterogeneity was substantial, and subgroup analyses were not feasible due to inconsistent reporting. Finally, although publication bias was assessed, undetected bias cannot be fully excluded. Future studies could account for confounders either via subgroup analysis in a primary study or further sensitivity analyses in individual patient-level meta-analyses.

## 6 Conclusion

In our systematic review and meta-analysis, women had higher long-term mortality following CABG compared with men. It is the largest study to date of sex-specific differences in long-term mortality (≥7 years). These findings highlight persistent sex-based disparities in long-term outcomes and underscore the need for future patient-level studies to clarify underlying mechanisms and guide tailored interventions.

## CRediT authorship contribution statement

**David T. Zhang:** Writing – original draft, Validation, Methodology, Investigation, Formal analysis, Data curation, Conceptualization. **Simrat Dhaliwal:** Writing – review & editing, Investigation, Data curation. **Navindra Tajeshwar:** Writing – review & editing, Investigation, Data curation. **Gregg A. Stevens:** Writing – review & editing, Investigation, Formal analysis, Data curation. **Mohammed Al-Sadawi:** Writing – review & editing, Supervision, Methodology, Conceptualization. **Michael Tao:** Writing – review & editing, Supervision, Methodology, Investigation, Formal analysis, Conceptualization.

## Conflict of interest

None.

## Figures and Tables

**figs1:**