# Supplementary material for: Sex-related differences in long-term mortality after coronary artery bypass graft surgery: A systematic review and meta-analysis
Source: Int J Cardiol Cardiovasc Risk Prev. 2026 Feb 26;29:200611. doi: 10.1016/j.ijcrp.2026.200611 (PMC12961219; doi:10.1016/j.ijcrp.2026.200611)
Supplement: Multimedia component 1 [file mmc1.docx]

**Supplemental Table 1:** Risk of bias assessment of included studies

| **Study**  **(Year)** | **Design** | **Selection (4)** | **Comparability (2)** | **Outcome (3)** | **NOS Total** | **Overall Risk** |
| --- | --- | --- | --- | --- | --- | --- |
| Rahimtoola et al. (1993)^13^ | Observational | 3 | 1 | 3 | 7 | Low |
| Carey et al. (1995)^14^ | Observational | 3 | 1 | 3 | 7 | Low |
| Davis et al. (1995)^15^ | Observational | 3 | 1 | 3 | 7 | Low |
| Weintraub et al. (2003)^16^ | Observational | 4 | 1 | 3 | 8 | Low |
| Guru et al. (2006)^17^ | Registry | 4 | 2 | 3 | 9 | Low |
| Saxena et al. (2012)^18^ | Registry | 4 | 2 | 3 | 9 | Low |
| Schwann et al. (2012) ITA/RA^19^ | Observational | 3 | 1 | 2 | 6 | Moderate |
| Schwann et al. (2012) ITA/SV | Observational | 3 | 1 | 2 | 6 | Moderate |
| Dalen et al. (2019)^20^ | Registry | 4 | 2 | 3 | 9 | Low |
| Vrancic et al. (2019)^21^ | Observational | 3 | 1 | 2 | 6 | Moderate |
| Piña et al. (2018)^22^ | RCT |  |  |  | Low | Low |
| Gaudino et al. (2020) single vessel^23^ | Registry | 4 | 2 | 3 | 9 | Low |
| Gaudino et al. (2020) multiple vessels | Registry | 4 | 2 | 3 | 9 | Low |
| Kyto et al. (2020)^24^ | Registry | 4 | 2 | 3 | 9 | Low |
| Friedrich et al. (2020)^25^ | Observational | 3 | 1 | 2 | 6 | Moderate |
| Hara et al. (2020)^26^ | Observational | 3 | 1 | 2 | 6 | Moderate |
| Sattartabar et al. (2021)^27^ | Registry | 4 | 2 | 3 | 9 | Low |
| Rubens et al. (2022)^28^ | Registry | 4 | 2 | 3 | 9 | Low |
| Nurkkala et al. (2022)^29^ | Registry | 4 | 2 | 3 | 9 | Low |
| Ram et al. (2022)^30^ | Observational | 3 | 1 | 2 | 6 | Moderate |
| Abreu et al. (2022)^31^ | Observational | 3 | 1 | 3 | 7 | Low |

NOS = Newcastle–Ottawa Scale, RCT = randomized controlled trial
